# But Is Ageing Really All Bad? Conceptualising Positive Ageing

**DOI:** 10.3390/geriatrics10060151

**Published:** 2025-11-18

**Authors:** Miriam Sang-Ah Park, Blake Webber, Stephen P. Badham, Christian U. Krägeloh, Vincenza Capone, Anna Rosa Donizzetti, Mohsen Joshanloo, Szabolcs Gergő Harsányi, Monika Kovács, Emily Hellis

**Affiliations:** 1 School of Social Sciences, Nottingham Trent University, Shakespeare Street, Nottingham NG1 4FQ, UKemily.hellis2024@my.ntu.ac.uk (E.H.); 2 Faculty of Health and Environmental Sciences, Auckland University of Technology, Private Bag 92006, Auckland 1142, New Zealand; chris.krageloh@aut.ac.nz; 3 Department of Humanities, University of Naples Federico II, 80133 Naples, Italy; vincenza.capone@unina.it (V.C.); donizzet@unina.it (A.R.D.); 4 Department of Psychology, Keimyung University, 1095 Dalgubeol Boulevard, Dalseo-gu, Daegu 42601, Republic of Korea; mjoshanloo@gmail.com; 5 Department of Social Psychology and Intercultural Psychology, Institute of Psychology, Faculty of Humanities and Social Sciences, Károli Gáspár University, Bécsi Str. 324. Building V., H-1037 Budapest, Hungary; 6 Institute of Intercultural Psychology and Education, Faculty of Education and Psychology, Eötvös Loránd University, Kazinczy u. 23-27, H-1075 Budapest, Hungary; kovacs.monika@ppk.elte.hu

**Keywords:** positive ageing, well-being, quality of life, later life, cyclic model

## Abstract

Ageing literature, while growing in huge volume in the past decades, is still largely dominated by frameworks and topics of frailty and decline. A shift in attention to conceptualising ageing more holistically to include psychosocial and emotional aspects as well as subjective experience is much needed, in order to better account for the ageing (well) experience and processes in today’s times. There is a large portion of older adults with relatively good health. As life expectancy increases around the world, many older adults are living longer and healthier overall, often wishing for their lives to continue being active, meaningful, and fulfilling. With this changing demographic in mind, we argue for a framework of positive ageing. We define positive ageing as a subjective, intentional experience, which includes the multi-dimensional construction of ageing well. The notion of positive ageing has the potential to widen the scope of gerontological research and to help guide policy and intervention development. Furthermore, this conceptual framework and a cyclic model of positive ageing presented in the current work can effectively complement current models and practices of care in geriatrics by taking a more person-centred and holistic approach to understanding and managing health and well-being.

## 1. Introduction

Ageing is an inevitable, continuous process experienced by an increasing number of people worldwide as they live longer and reach old age. Between 2015 and 2050, the global population of those aged 60+ will almost double from 12% to 22% [1], and accordingly, the literature focusing on the topic of ageing and older adulthood is also seeing a substantial growth. However, ageing is most often associated with the decline and reduction in one’s functioning and abilities, with academic literature dominated by topics such as dementia, falls, depression, and poor physical health rather than a focus on the ‘good’ ageing.

While the emphasis on prevention of risks and improvement of care for this population is important, it is also worthwhile to shift the attention towards well-being and quality of life for older adults, acknowledging heterogeneity of those in the ‘aged’ category of the population. Different populations of older adults can be categorised by age groups (e.g., ‘young-old’, ‘old’, ‘old-old’), frailty, as well as general health conditions and ease with which they engage in activities such as work and travel [2]. What needs to be recognised is that there is a large portion of older adults with relatively good health, and as life expectancy increases around the world, many older adults are living longer and healthier overall, often wishing their lives to continue being active, meaningful, and fulfilling. It is with this focus on the overall well-being and quality of life of older adults that we discuss the importance of positive ageing, or ageing well, as a concept and an alternative framework for ageing research and practice.

## 2. Conceptual and Theoretical Foundations

### 2.1. Conceptualising Positive Ageing

As a relatively novel concept, positive ageing has yet to have a formal and recognised definition [3,4]. The proliferation of interest in the concept is thought to have begun with Rowe and Kahn’s [5] successful ageing [6], defined as avoidance of disability and disease, having high cognitive and physical capabilities, and an active engagement in life. However, this definition has since been heavily criticised, primarily for its physiological emphasis [7] and seeming disregard of social and psychological factors [8,9]. It has also been criticised for its emphasis on individual responsibility [10] and neglect of cultural diversity [11]. These limitations have resulted in the inability to recognise a significant proportion of older adult populations as successful agers [6,12,13,14,15].

Consequently, several other discipline-dependent concepts have been developed (Table 1), such as ageing well [16], healthy ageing [17], and productive ageing [18], all of which refer to different elements of an older adult’s quality of life [19]. However, many of these ageing concepts lack a holistic view on health, have a cultural bias, place too much emphasis on physical and psychological functionality, and therefore may fail to explain diverse experiences of older adults from different demographic backgrounds. Given the discipline-specific, uni-dimensional, and narrow conceptualisations of ageing, there has been a call for a better model, such as one that especially takes into account the perspectives and lived experiences of older adults [20,21]. Although there is confusion pertaining the predictors and their outcomes [22], numerous factors have been identified as important for ageing well by older adults.

Positive ageing can be thought of as an emerging paradigm in gerontology [22] for the reasons that called for its conceptualisation in the first place [5]. Positive ageing is an attempt to refocus the understanding of ageing from one marked by loss, pathology, disability, and burden, to that of resilience, fulfilment, meaning, and positivity [24,25,26]. It aims to capture a favourable ageing process and regard older adults and their experiences holistically [27], incorporating a myriad of biopsychosocial factors which former and earlier ageing concepts may not have been able to capture effectively [28]. It allows a focus on the sociocultural context and its role in shaping the ageing experience (e.g., societal perceptions of older adults, welfare provisions, opportunities to engage in civic matters), as well as the subjectivity of the ageing experience. As Kornadt et al. [29] demonstrate, one’s subjective views on ageing are a powerful predictor of ageing outcomes, with more favourable self-perceptions of ageing leading to better health and ageing outcomes overall. We may therefore define positive ageing as a “subjective, intentional experience and multi-dimensional construction of ageing well” [30], and argue this to be a more comprehensive and useful framework for understanding ageing processes and outcomes.

Indeed, several reviews of the literature have also revealed that older adults consider positive ageing as a multi-dimensional concept, with biological, psychological, and social components [3,22,31,32,33,34]. Biological components encompass aspects of good physical health, maintenance of physical and cognitive functioning, and being free from disability and illness [3,31,32]. Psychological components include features such as acceptance of old age, maintenance of independence, high quality of life, and a positive attitude or mindset [3,31]. Social factors comprise participating in social activities, relationships with family and friends, and other themes around engaging in the local community and wider society [3,31,33,35]. Most prevalent across cultures [32] and amongst lay people and academics [3] is social engagement, and the role of the family might be of particular relevance in positive ageing. In an open-ended question of what makes life most meaningful, family was most frequently mentioned across all age groups in 14 out of the 17 countries [36]. This finding is interesting in the sense that the significance of family and strong engagement and connection with the family have traditionally gained less attention in Western cultures, with the emphasis on independence and autonomy. However, with the growing awareness of the detrimental impact of loneliness and social isolation on older adults’ health and well-being [37,38], the attention has shifted over to meaningful social ties and relationships, including those with one’s family members. Park et al.’s study of older adults in the UK [30] found that interconnectedness and living harmoniously with others were important for ageing positively, which calls for further research on the role of close relationships in older adults’ well-being globally.

### 2.2. Positive Ageing: Expansion and Application

There are a few ways in which the concept of positive ageing can be expanded and applied to research and practice aimed at improving older adults’ health and well-being. Firstly, we discuss how future research applying positive ageing can look deeper into the construction of meaningful life in old age from the individuals’ point of view. We then consider implications for research including ageing measures used in contemporary research.

Relatively little is known about what makes a meaningful and satisfying life in old age, and one finds a lack of consensus in the literature on what it constitutes. Aside from the more obvious factors such as financial security and good physical health, there must be more subjective views and opinions about what makes a good life that apply to daily functioning and future planning in old age. Considering that one often adheres to guiding principles in their lives, such as priorities and life goals, it may be useful to explore the philosophical foundations for meaningful life and well-being (e.g., [39]). For instance, for those who value generativity, or the desire to give something back to the others and the society, a life in old age that involves giving (e.g., volunteering, helping with family needs such as babysitting or financial support) may render happiness and fulfilment, leading to better well-being overall even if they struggle with other aspects of life [30]. As such, some of the unique concepts that may be especially pertinent to looking at a meaningful life in old age need to be explored further. Cultural and cross-cultural exploration of the contributing factors to positive ageing would be especially useful here, as the construction of positive ageing as influenced by general world views and priorities in life will differ across cultures. For instance, we may observe that cultures with an emphasis on autonomy versus relatedness render differences in the balance of priorities in terms of independence and interdependence [40]. All in all, these varying cultural conceptions of ageing impact what is perceived as a fulfilling or meaningful life in older age and thus need to be understood within a global context. As Moody’s review recommends [41], there is value in illuminating the role of specific faith and the generated meanings that lead to successful ageing for different individuals. Religion, philosophical underpinnings, and cultural norms are important to consider as they will have shaped such constructions and can give us insights on how to guide older adults in specific cultural spheres to age more positively. For example, religious frameworks in different traditions (e.g., Christianity, Buddhism, Hinduism) may offer distinct paths to fulfilment in old age, including spiritual practices, the pursuit of wisdom, or community involvement, which can provide individuals with a sense of purpose and meaning that transcends physical health or financial security. The intersection of religion and cultural values creates different narratives about the “good life” that may guide elderly individuals in their later years, making it essential to consider these dimensions when discussing ageing [42].

In particular, we highlight the potential significance of values such as gratitude and empathy for positive ageing. Also, as aforementioned, concepts such as generativity and dialecticism, which may be relatively novel ideas strongly grounded in Eastern philosophy, can be applied. Most of these concepts emphasise interconnectedness rather than individualism in ageing, and also calls for a more interdisciplinary approach. Gratitude, for example, fosters emotional well-being and life satisfaction, especially in old age when one has to deal with loss or decline. Empathy strengthens social bonds and mental health, while dialecticism encourages accepting life’s contradictions, helping to navigate ageing’s challenges. These ideas promote a holistic view of ageing, integrating emotional, relational, and philosophical dimensions, and call for an interdisciplinary approach that combines psychology, sociology, and philosophy to understand both the material and immaterial aspects of ageing. This broad set of ideas emphasises a need for researchers to undertake a formal interdisciplinary-focused concept analysis of positive ageing founded by established approaches such as [43,44].

Furthermore, there are other individual-level factors to consider for positive ageing. Potential differences in priorities and views towards positive ageing by one’s socioeconomic as well as cultural background also deserve further attention. Those from higher socioeconomic backgrounds typically have access to better healthcare, social activities, and a higher standard of living, which can enhance their well-being and shape their view of a fulfilling old age. In contrast, individuals from lower socioeconomic backgrounds may face challenges like economic insecurity, social isolation, and limited healthcare, affecting their quality of life [45]. These disparities impact not only available opportunities but also perceptions of a meaningful life and should be accounted for when constructing frameworks for positive ageing to ensure inclusivity and applicability across various populations. Policymakers, healthcare professionals, and community organisers must recognise existing socioeconomic inequalities and work toward solutions that bridge the gap, ensuring all older adults, regardless of financial standing, have access to opportunities for meaningful ageing.

Measurement and indicators of ageing are also heavily reliant upon biomedical underpinnings, which tend to overlook other important dimensions of one’s health and well-being, such as psychosocial aspects and subjective experience. As Park et al. [46] argue, perceiving ageing as a ‘decline’ in functions is clearly reflected in some of the existing measures, and some may even include ageist assumptions and wording. When one looks at the population of older adults now, it is clear that they do not only live longer, but they also tend to be healthier and have a higher level of functioning ability overall compared to what previous generations saw. Badham’s review [47] demonstrates the trend in scientific data that finds improvement in the cognitive ability of older adults over the years and past decades, which provides some evidence for this argument. Carver and Buchanan [48] testify to the multi-dimensional nature of the concept, with the findings from their review highlighting the need for ageing to be viewed as a complex process that requires a holistic perspective. As such, ageing measures need to better reflect the contemporary ageing experience of older adults and also take into account the more subjective views, including positive aspects (e.g., [46]) and holistic understanding of health and well-being. While many existing studies rely mostly on objective health measures—such as disease prevention, cognitive health, and maintaining physical abilities [1]—to gauge good ageing, there are critical gaps in these approaches that fail to capture the broader experience of positive ageing [49]. For instance, it can be pointed out that the Active Ageing Index, a popular tool developed for European countries for monitoring active ageing, fails to take account of capabilities, resources, and preferences, which are just as important indicators for one’s potential ageing outcomes. Addressing these shortcomings requires a paradigm shift toward interventions that integrate psychological well-being, social engagement, physical activity, and a sense of purpose. Park et al.’s Positive Ageing Scale [46] takes a step forward in this approach, encapsulating the meaning and value of positive ageing and taking into account one’s own evaluation of his/her own ageing experience.

Existing initiatives, such as population health management programmes which come from a background focused on successful and active ageing definitions, either primarily focus on specific health behaviours such as smoking and alcohol usage [50], physical activity [51], risk of falls [52], or age-related diseases such as dementia [53] through randomised controlled trials. The necessarily controlled and reductionist nature of such studies does not account for the holistic process of positive ageing or diversity of individuals, emphasising the rhetoric of frailty and disease. Interventions based on more holistic perspectives on health and well-being consider the heterogeneity of the older adult population and help to inform more effective, long-lasting interventions for ageing populations. Interventions focusing on encouraging older adults to adopt a more positive subjective view of ageing could be one such example, where more positive attitudes and perceptions towards one’s ageing process can promote behaviours that can support positive ageing [54].

### 2.3. Cyclic Model of Positive Ageing

**Figure 1:**
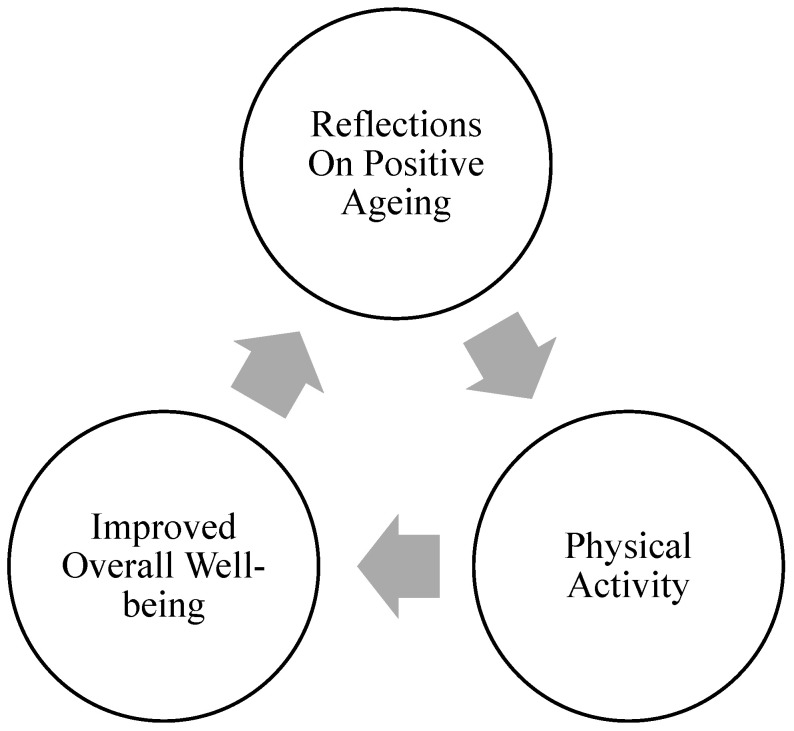
The cyclic model of positive ageing.

For better understanding of the dynamics of positive ageing processes, we suggest a cyclic model, emphasising the interconnected relationship between mindset, physical activity, and overall well-being (Figure 1). Physical activity has been shown to reduce the risk of age-related diseases, improving health outcomes such as lower mortality, reduced falls, and decreased cognitive decline [55,56,57]. Similarly, a positive mindset and engaging with reflections about the positive aspects of ageing could also lead to better well-being. Positive mindset, which may be linked to mindfulness, has indeed been associated with enhanced mental health, well-being, and successful ageing [58]. This model suggests that if older adults engage in positive ageing reflections and adopt a positive mindset, they are more likely to also engage with physical activity, and vice versa. The combination and interplay between these elements lead to improved overall well-being, which reinforces positive reflections on ageing, creating a continuous cycle of growth and health.

The cyclic model of positive ageing can be integrated into interventions in several ways. Given the broad positive health outcomes associated with self-reflection (see [59] for a summary), encouraging reflection through journaling, self-reflection exercises, and workshops could help cultivate a positive ageing mindset. Mindfulness-based traits can help improve psychological well-being [58] activities, and mindfulness-based stress reduction, meditation, and creative practices like art and nature therapies may therefore be a crucial component of positive ageing. Increasing physical activity has clear benefits for health in older adults (e.g., see [60] for a review) and can be achieved through enjoyable, social activities like yoga, walking groups, and also exercise programmes incorporating mindfulness, such as tai chi. To be effective, interventions should involve older adults in the design process, emphasise strengths and opportunities, and leverage technology to create accessible platforms, supporting fuller, more satisfying lives for older adults. Most importantly, interventions based on the cyclic model will encourage the participants to work on their health more holistically and allow them the opportunity to develop more positive attitudes towards ageing. Such interventions could include structured programmes which combine physical activity, mental health practices, and social engagement in a way that reflects the interdependent cycles of well-being. For example, an intervention may combine exercise with reflective practices, such as that seen in Yi et al.’s intervention which uses Qigong practice and forest therapy walking [61], or involve group activities which strengthen social connectedness, cognitive resilience, and good mental health, such as Chi, Liu, and Wu’s ‘Active Aging’ intervention [62]. These positive attitudes per se are likely to improve their overall functioning and lead to better ageing outcomes.

## 3. Conclusions

As the global population of older adults continues to grow and live longer, it becomes increasingly crucial to shift the narrative around ageing from one of decline and loss to one of resilience, fulfilment, and well-being. The current work refines the definition of positive ageing, that has taken different forms across multiple disciplines, and proposes an interdisciplinary consideration of ageing as a holistic process of biological, psychological, and social health. Positive ageing offers a comprehensive, person-centred framework that emphasises the multifaceted nature of ageing, integrating biological, psychological, and social components. The cyclic model of positive ageing further enriches this framework by highlighting the interconnected relationship between mindset, physical activity, and overall well-being. This model suggests that fostering a positive ageing mindset, along with encouraging physical activity, creates a continuous cycle of growth and health that enhances overall well-being. At the core of this conceptual shift is our definition of positive ageing as a subjective and intentional experience and as a multi-dimensional construction of ageing well, which underscores the importance of understanding ageing as a personalised, dynamic process influenced by individual perspectives, choices, and experiences. By moving beyond traditional models that focus solely on physical health and disease prevention, positive ageing fosters a more holistic understanding of what it means to age well. Furthermore, integrating this framework into research and policy can lead to more inclusive interventions, ensuring that older adults, regardless of their health status or background, are supported in achieving meaningful, active, and fulfilling lives. As we continue to explore and expand the concept of positive ageing, it is essential to focus on resilience, purpose, and community engagement—factors that can significantly enhance the quality of life for older adults, now and in the future.

## Figures and Tables

**Table 1:** Ageing definitions.

Concept	Definition	Components	Advantages	Disadvantages
Successful Ageing	High physical, psychological, and social functioning in old age without major diseases [5]	Physical health Psychological health Cognitive functio	Clear measurable components Focus on prevention Encourages social engagement	Excludes those with chronic illness or disabilities Cultural bias towards Western norms Lack of recognition of social and economic barriers Narrow focus. A potential to stigmatise
Active Ageing	The process of optimising opportunities for health, participation, and security, in order to enhance quality of life as people age [23]	Overall health Quality of life Participation Security	Focus on quality of life	Assumes equal access to opportunities, may potentially exclude vulnerable groups
Healthy Ageing	The process of developing and maintaining the functional ability that enables well-being in older age [17]	Physical health Psychological health Well-being	Personal empowerment—encourages individuals to take control of their ageing process	Places too much emphasis on functionality
Ageing Well	Continuing to live in the community with good physical and psychological health [16]	Physical health Psychological health Social relationships	Highlights the importance of social connections	Emphasis on physical function and psychological health, being free from disability and disease
Productive Ageing	The involvement of older adults in activities that contribute to their own well-being and the well-being of others or society. including paid and unpaid work, volunteering, and social participation [18]	Physical health Social relationships	Economic and social benefits	Excludes activities such as physical exercise, intellectual, and spiritual activities Cultural bias to Western ideals

## Data Availability

Not applicable.
